# INS-fOCT: a label-free, all-optical method for simultaneously manipulating and mapping brain function

**DOI:** 10.1117/1.NPh.7.1.015014

**Published:** 2020-03-31

**Authors:** Ying Zhang, Lin Yao, Fen Yang, Shanshan Yang, Akshay Edathodathil, Wang Xi, Anna Wang Roe, Peng Li

**Affiliations:** a Zhejiang University, College of Optical Science and Engineering, State Key Lab of Modern Optical Instrumentation, Hangzhou, China; b Zhejiang University, Interdisciplinary Institute of Neuroscience and Technology, School of Medicine, Hangzhou, Zhejiang, China; c Zhejiang University, Key Laboratory of Biomedical Engineering of Ministry of Education, Hangzhou, Zhejiang, China; d Oregon Health & Sciences University, Oregon National Primate Research Center, Division of Neuroscience, Beaverton, Oregon, United States; e Zhejiang University, International Research Center for Advanced Photonics, Hangzhou, Zhejiang, China

**Keywords:** functional optical coherence tomography, infrared neural stimulation, functional imaging

## Abstract

**Significance:** Current approaches to stimulating and recording from the brain have combined electrical or optogenetic stimulation with recording approaches, such as two-photon, electrophysiology (EP), and optical intrinsic signal imaging (OISI). However, we lack a label-free, all-optical approach with high spatial and temporal resolution.

**Aim:** To develop a label-free, all-optical method that simultaneously manipulates and images brain function using pulsed near-infrared light (INS) and functional optical coherence tomography (fOCT), respectively.

**Approach:** We built a coregistered INS, fOCT, and OISI system. OISI and EP recordings were employed to validate the fOCT signals.

**Results:** The fOCT signal was reliable and regional, and the area of fOCT signal corresponded with the INS-activated region. The fOCT signal was in synchrony with the INS onset time with a delay of ∼30  ms. The magnitude of fOCT signal exhibited a linear correlation with the INS radiant exposure. The significant correlation between the fOCT signal and INS was further supported by OISI and EP recordings.

**Conclusions:** The proposed fiber-based, all-optical INS-fOCT method allows simultaneous stimulation and mapping without the risk of interchannel cross-talk and the requirement of contrast injection and viral transfection and offers a deep penetration depth and high resolution.

## 1 Introduction

In the study of cortical function, numerous technologies (electrical, optical, magnetic, ultrasound, and chemical methodologies) are now available for modulating and recording neural function. As briefly summarized below, each has its advantages and limitations. However, currently no method is available that couples contact-free focal stimulation with high-spatial resolution, depth-resolved, contact-free monitoring of cortex at mesoscale (submillimeter to millimeters scale). Moreover, technologies appropriate for mice (e.g., transgenic mice) require interventions that are less easily used in large animal models. Here, we have developed a label-free, all-optical imaging, and stimulation approach for rodent model, which is broadly applicable for other animal models theoretically.

For recording, single unit electrophysiology is the gold standard of monitoring neural function. However, sampling is limited by electrode recording site geometries and requires insertion of electrodes into the brain. Two-photon imaging provides dense sampling and cellular resolution in x, y, and z axes, but it is limited by sampling areas (∼300 to 600  μm fields of view) and requires labeling of cells with injection of dyes^1^^,^^2^ or transgenic methods.^3^^,^^4^ Three-photon imaging may achieve depths of 1 mm or more in rodents^5^^,^^6^ but is limited in the imaging speed due to its intrinsic low quantum mechanical efficiency. Recording with GRIN lenses involves insertion of large mm-sized probes that cause considerable tissue damage.^7^ Optical imaging with voltage-sensitive dyes (VSD) provides large-scale, high temporal resolution (1 to 10 ms) mapping.^8^^,^^9^ But in large animals, the difficulties associated with VSD tissue staining and photodynamic damage have limited its popularity. Optical intrinsic signal imaging (OISI),^10^ based on hemodynamic signals (initial dip), is a common method for imaging at larger scale and can be conducted without application of substances into the brain. OISI signals correlate well with neuronal population response and are useful for mapping cortical columns. However, OISI is unable to resolve signals from different laminar depths within cortical tissue.

Optical coherence tomography (OCT) monitors neural activity based on changes in the intrinsic optical scattering properties of neural tissue with changes in membrane potential.^11^^–^^15^ The intrinsic optical scattering signal is linearly proportional to the change in the membrane potential in cultured neurons^15^^,^^16^ but is exceedingly small in magnitude.^17^ OCT, an optical interferometric technique, is capable of recording depth-resolved scattering information by sending a wave into the sample and then measuring the echoes reflected from tissue scattering. OCT offers micrometer-level resolution and a sensitivity exceeding 100 dB over a subsurface depth of 1 to 2 mm into intact brain tissue, making it well suited for detecting the small changes of intrinsic scattering from the cerebral cortex. The fractional change of OCT scattering signal, usually termed as functional OCT (fOCT), has been used to map the functional response to visual stimulation in cat cortex^18^^–^^20^ and the response to electrical stimulation in rat cortex.^21^^,^^22^ Graf et al.^23^ observed a direct correlation between change in membrane voltage and fOCT scattering intensity during action potentials in a single cultured Aplysia bag cell neuron. Thus, fOCT is a potentially attractive tool for detecting electrical activity in neural tissue *in vivo*.^18^^–^^27^

For many studies (e.g., aimed at neuromodulation or functional tract tracing), neural stimulation methods are desirable. Electrical stimulation has been a long-standing tool for neurostimulation,^28^^,^^29^ but it suffers from spread of electrical current, often leading to unwanted activation of additional brain circuits and resulting side effects.^30^^,^^31^ In addition, stimulation artifacts make simultaneous electrophysiological stimulation and recording difficult. Development of optical approaches has introduced distinct advantages. Optogenetics is an exciting cell-type specific stimulation approach that is compatible with electrical recording in anesthetized and awake behaving animals.^3^^,^^7^^,^^32^ However, in animals such as primates, it requires injection of viruses and time for viral expression (typically 4 to 6 weeks); moreover, optical stimulation sites are limited only to the sites of viral expression. Other larger scale stimulation methods are available (transcranial magnetic stimulation and ultrasound), but their spatial resolution is relatively low.^33^^–^^35^

A relatively new stimulation technology for study of cortical function *in vivo* is infrared neural stimulation (INS).^36^^,^^37^ This method has been demonstrated to activate cortical neurons as assessed by electrophysiological recordings,^38^^–^^40^ optical calcium imaging,^41^ OISI,^42^ and fMRI.^43^ Using trains of pulsed infrared light delivered via a fiber optic (e.g., 100  μm to 1 mm in diameter), INS leads to membrane capacitance change and depolarization of the neuronal membrane.^36^^,^^38^^,^^40^^,^^44^ Although not cell-type specific, INS achieves high spatial precision of stimulation (matching the size of fiber optic used). Importantly, it is not dependent on viral expression, thus permitting application of stimulation anywhere in the brain (for review see Ref. 45). This stimulation method is safe and effective within specific ranges of stimulation intensity, frequency, and duration.^42^^,^^44^^,^^46^

Many recording and stimulation methods have above been used in different combinations. However, none are contact-free (do not require insertion or application of any materials), large scale (mm to cm scale), depth resolvable (can distinguish different depths), and readily applicable to large animal models. To achieve such a capability, we have combined two optical methods, OCT and INS. We demonstrate a label-free, all-optical approach for manipulating and mapping brain function in a contact-free, large-scale, depth resolvable manner in cerebral cortex up to a millimeter in depth.

## 2 Materials and Methods

### 2.1 Animal Preparation

Male Sprague–Dawley rats (n=5; 300 to 500 g) were anesthetized with 1% pentobarbital sodium (Sigma, P3761-25g). The toe-pinch test was used to ensure the animal was in an adequate state of anesthesia. The animal was placed in a stereotactic frame and a craniotomy and durotomy were performed to expose somatosensory cortex (+2 to −3  mm anterior/posterior and 4 mm lateral to bregma). Mannitol (1.0 ml, 20% concentration) was given to prevent potential brain edema. Warm (∼37°C) 1.5% agar in saline was used to stabilize the cortex and a glass coverslip was placed on the agar to create an optical imaging window ($∼4\times 3\;{\mathrm{mm}}^{2}$). Dental cement was used to seal the cranial window to the skull. All animal experimental procedures used in this study were approved by the Animal Care and Use Committee of Zhejiang University.

### 2.2 Experimental System and Data Acquisition

**Fig. 1:**
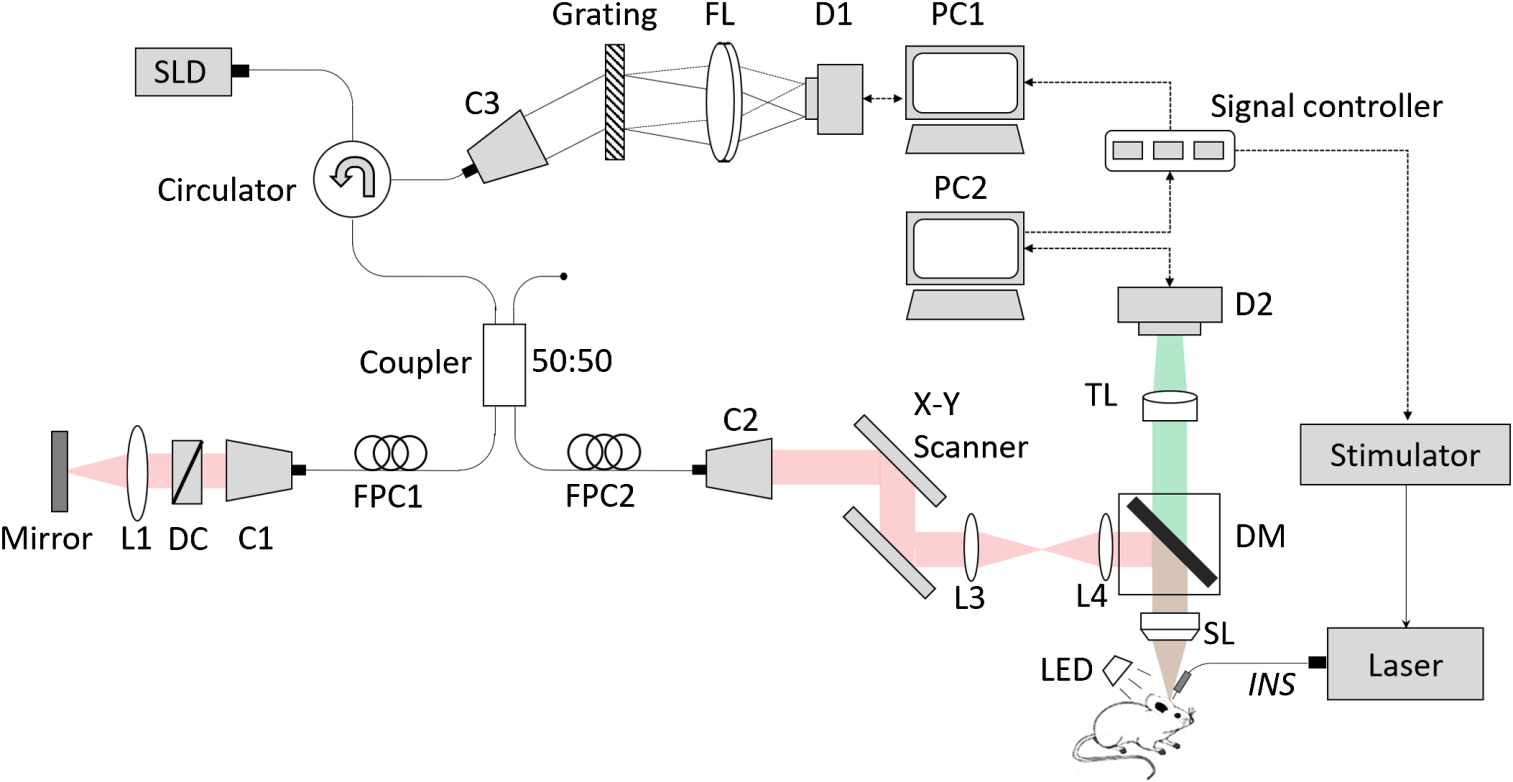
Schematic of the coregistered INS and fOCT and OISI system. L, lens; C, collimator; D1, InGaAs camera; D2, CCD camera; DC, dispersion compensator; PC, personal computer; FPC, fiber polarization controller; FL, Fourier lens; TL, tube lens; SL, scanning lens; SLD, super luminescent diode; DM, dichroic mirror; LED, 540/632-nm LED. Inset: The INS schematic. Each trial is composed of 1-s prestimulus, 0.5-s stimulation, and 18.5-s poststimulus. INS pulse parameters: pulse width=250  μs, pulse repetition rate=200  Hz. A total of 15 trials were applied.

Simultaneous OISI and OCT were performed on rat somatosensory cortex. Figure 1 shows the schematic of the coregistered INS and fOCT and OISI system. OCT is primarily based on a typical Fourier-domain configuration. The superluminesent diode had a radiant power of 10 mW, a central wavelength of 1325 nm, and a full-width at half-maximum bandwidth of 100 nm, theoretically offering an axial resolution of ∼7.6  μm in air. The output light was delivered into a 2×2 fiber coupler and split into the reference and sample arms, respectively. The OCT detection unit was a high-speed spectrometer equipped with a fast line-scan InGaAs camera (Sensors Unlimited Inc., SU1024-LDH2, 92 kHz line-scan rate, and 1024 active pixels). The camera output was digitalized and fed to a custom-designed program in PC1. In the OCT sample arm, an x-y galvanometer was adopted for three-dimensional (3-D) volume scanning. A scanning lens (Thorlabs, LSM03) with an effective focal length of 36 mm was used to collimate the detecting light on the sample, yielding a lateral resolution of ∼10  μm. Prior to the scanning lens, a dichroic mirror (Semrock, FF700-SDi01) was employed to separate the light paths for coregistered OCT and OISI. The OISI system employed 540-nm green LED illumination for acquiring cortical blood vessel distribution map and 632-nm red LED illumination for observing changes in blood oxygen concentration. Light reflected from the cortex transmitted through the dichroic mirror and a tube lens onto a CCD camera (Photonfocus, MV1-D1312-160, 1312×1082  pixels). The coregistered OISI-OCT had a field of view of ∼3×2.5  mm in the x−y plane. OCT offered an imaging range of 2.5 mm in the depth (z) direction. The INS-OCT and OISI system were synchronized by signal controller, which is triggered by PC2.

In this study, the volumetric OCT dataset (z−x−y) was acquired with a raster scanning protocol. In the fast-scan (x) direction, 512 A-lines formed a B-frame. A total of 420 B-frames were acquired with equal interval in the slow-scan (y) direction. To record the time course of INS-evoked responses, OCT was then switched to a B-M mode scanning protocol (i.e., disabling the slow-scanner for repeated B-scan). In this study, the OCT B-frame rate was set to be 100 frames per second (fps), and OISI frame rate was set to be 20 fps. For temporal analysis (Fig. 4), the frame rate of both was increased to 240 and 60 fps, respectively.

### 2.3 Laser Stimulation Protocol

INS was performed using a near-infrared diode laser at 1870±20  nm wavelength (Cygnus Technology Inc., PG4000A). Such a wavelength was selected for reasonable energy transfer and reasonable tissue penetration of 300 to 600  μm.^47^ Light was delivered through a fiber (200  μm diameter and 0.22 numerical aperture) onto the region of interest, yielding a focal stimulation. Each INS pulse had a 250-μs pulse width and pulse trains were delivered at a repetition rate of 200 Hz. Each trial consisted of a 1-s prestimulus period, followed by 0.5 s INS (i.e., a train of 100 pulses) and a subsequent 18.5-s poststimulus period that allowed full recovery to baseline, as showed in the inset schematic in Fig. 1. A total of 15 trials were repeated. Four different radiant exposures (i.e., 0.3, 0.5, 0.7, and $1.0\;J/{\mathrm{cm}}^{2}$ per pulse) were selected for radiance dependency study. In each experiment, an additional blank (no stimulation) condition was imaged.

### 2.4 Data Analysis

The raw OCT cross-sectional images were reconstructed for each trial, which could be denoted as a 3-D array I(z,x,t) with z is the depth direction, x is the lateral direction, and t is the temporal dimension. The prestimulus baseline value $I_{b}$ is determined by averaging intensity over the prestimulus period $t_{1:N}$:

$$I_{b}(z,x)=\frac{1}{N}\overset{N}{\underset{i=1}{\sum }}I(z,x,t_{i}),$$ (1)

where i is the index of B-frame, and N corresponds to the frame number obtained during the prestimulus period (−1 to 0 s in this study).

To rule out the spontaneous intensity fluctuations and improve the SNR of OCT signals, an adaptive processing algorithm^48^ was employed to emphasize significant pixels. The pixel $\left(z,x,t_{i}\right)$ was a positive significant signal pixel if its intensity value was greater than the baseline value $I_{b}$ plus three times the prestim standard deviation (STD) $3\sigma_{b}(z,x)$ in five continuous frames:

$$I(z,x,t_{i}:t_{i+4})>I_{b}(z,x)+3\sigma_{b}(z,x).$$ (2)

Similarly, the pixel $\left(z,x,t_{i}\right)$ was a negative significant signal pixel if its intensity value was less than the baseline value $I_{b}$ minus $3\sigma_{b}$ in five continuous frames:

$$I(z,x,t_{i}:t_{i+4})<I_{b}(z,x)-3\sigma_{b}(z,x).$$ (3)

**Fig. 2:**
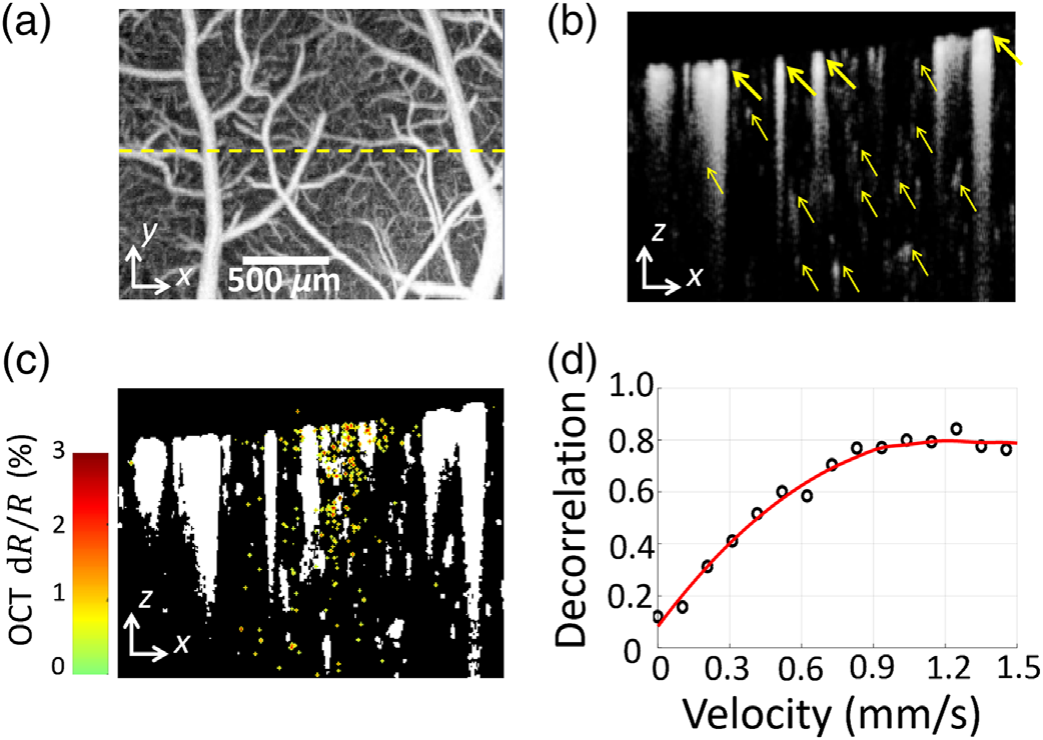
Binary vascular mask processing and flow velocity measurement. (a) Projection view of OCT angiogram. The dashed yellow line indicates the locus of fOCT scan. (b) Cross-sectional OCT angiogram (gray) along the dashed line in (a). Bold arrows indicate the large blood vessels, which are mainly located in the superficial pial layer. Thin arrows indicate the capillary blood vessels, which are mainly located in the cortical layer. (c) Vascular binarization mask (white areas represent blood vessels and tail artifacts) superimposed with OCT cross-sectional activation map (color) from INS. (d) Plot of the measured decorrelation values versus the velocity of the flow phantom. The phantom velocity is controlled by a syringe pump.

To further exclude the hemodynamic influence on fOCT signals, a binary vascular mask was applied to the OCT raw data. OCT angiogram was introduced in Figs. 2(a) and 2(b) to emphasize blood flow by mathematically analyzing the temporal dynamics induced by moving red blood cells (RBCs).^49^ The cross-sectional angiogram was binarized to generate an avascular mask for each frame in one trial, where the vessel area was set to be zero and the surrounding tissue was one. The intersection of all single-frame avascular masks was taken as the final vascular mask, as showed in Fig. 2(c). Inter-B-scan decorrelation value was computed for velocity quantification on a basis of previous studies.^50^ And we confirmed that the calculated decorrelation value monotonically increases with the velocity of flow phantoms within a range from 0 to 1.2  mm/s, as shown in Fig. 2(d). The preparation of the flow phantoms has been detailed in Ref. 51.

After applying both the adaptive processing and the avascular masks, the fractional change of OCT scattering signal dR/R is defined as the relative ratio of the poststimulus frames over prestimulus baseline value:

$$\frac{dR}{R(z,x,t_{i})}=\frac{I(z,x,t_{i})-I_{b}(z,x)}{I_{b}(z,x)}.$$ (4)

The fOCT signals were averaged over all the significant pixels within the whole cross-sectional frame (3×2.5  mm size in the x–z plane) and 15 repeated trials for noise reduction, and the pixels without sufficient intensity were excluded with an intensity mask using an intensity threshold of six times STD above the noise level. The filtered negative responses were inversed to be normalized with the positive ones and the final significant fOCT signals were generated by their averaging. To see the hemodynamic response to INS, the RBCs-induced interframe decorrelation value of OCTA was used as the velocity index of blood flow.^49^

OISI and OCT were spatially coregistered using pial blood vessels as rough landmarks and shifting the ROI until Pearson’s correlation coefficient of OISI image (540-nm green LED illumination) and projection view of OCT reached the maximum. Accordingly, the functional OISI signals were calculated in a similar way yet without masks.^42^ Relative intensity changes to prestim baseline for each pixel were calculated and averaged within ROI as functional OISI signal in response to INS.

### 2.5 Electrophysiology Recordings

Electrophysiology recording was also employed to validate that INS evoked neuronal activities successfully. Single-unit and multi-unit electrophysiology was utilized to evaluate the rat cortical neuronal responses within 30 trials of the INS stimulus protocol (i.e., 1-s prestimulus period, followed by 0.5 s INS and a subsequent 18.5-s poststimulus period). Glass-coated tungsten microelectrodes (∼1.3  MΩ) were inserted into the rat cortex at depths of 50 to 750  μm in somatosensory regions of interest. The fiber optic was placed ∼500  μm away from the microelectrode. The sampling rate is 30 kHz. Signals were recorded using a Cerebus system (Blackrock Microsystem, LB-0028-14.00, 128 channels), and peristimulus time histograms (PSTHs) were generated and analyzed using Matlab software. The paired two-tailed t test was employed to validate the statistical significance of the INS-evoked activation change.

## 3 Results

### 3.1 Spatial Analysis

**Fig. 3:**
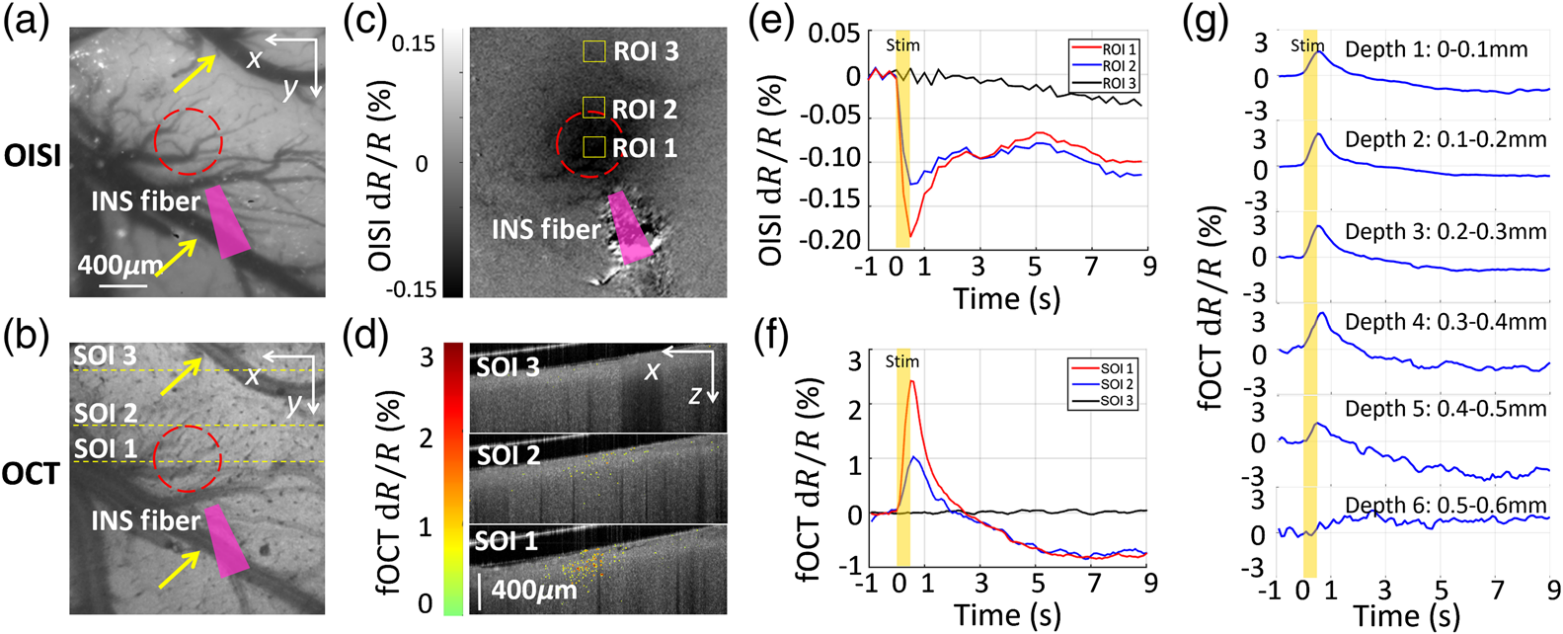
Spatial correspondence of INS-evoked fOCT signals in rat cortex. (a) OISI blood vessel map with 540-nm green light illumination. (b) Projection view of the 3-D OCT structural image. Pearson’s correlation coefficient of (a) and (b)  = 0.85. Yellow arrows in (a), (b) indicate pial blood vessels. Red circles in (a), (b) indicate site of INS stimulation. ROI means region of interest in OISI. SOI means section of interest in OCT. ROI 1 and SOI 1, sites at INS center; ROI 2 and SOI 2: sites near INS edge; ROI 3 and SOI 3, sites distant from INS. The closer to the INS center, the stronger the signals. The yellow dashed lines indicate SOIs 1 to 3 in fOCT. (c) OISI activation map in response to INS with 632-nm red light illumination at time window t=0.5  s. Darkening indicates activation. The yellow boxes indicate ROIs 1 to 3 (20×20  pixels) in OISI. (d) fOCT cross-sections (green-red color scale) superimposed with the OCT anatomical image (gray scale) at time window t=0.5  s. (e) Averaged OISI time-courses from ROIs 1 to 3 in (c). For ROI 1, dR/R=0.12±0.018% (mean±STD of 15 trial) at 0.5 s. (f) Averaged fOCT time-courses from SOIs 1 to 3 in (d). For SOI 1, dR/R=2.5±0.52% (mean±STD of 15 trial) at 0.5 s. (g) Depth-resolved, averaged fOCT time-courses from depths 1to 6, i.e., 0 to 600  μm under the superficial cortex, of SOI 1 in (d). Each depth contains a thickness of 100  μm.

To characterize the INS-evoked fOCT signal, we examined the spatial and temporal profiles of response, as well as its intensity dependence. As we have previously examined INS-induced neural response using 632-nm OISI, we used this as a standard of comparison. As shown in Figs. 3(a) and 3(c), INS ($1\;J/{\mathrm{cm}}^{2}$) applied to rat cortex evokes a localized OISI signal (a darkening of tissue due to neural activity induced deoxygenation) roughly 0.4 mm in diameter. As shown in Fig. 3(e), maximal optical reflectance changes were observed at the INS center (ROI1). INS-induced response dropped by roughly a third near the periphery of the stimulation zone (ROI2) and was still near baseline outside (ROI3) the locus of stimulation. Moreover, the INS-induced response was in synchrony with the stimulus and reached a peak on the order of ∼0.2% (relative intensity change), consistent with functional hemodynamic changes described in previous studies.^42^

We then evaluated whether OCT could detect similar signatures of functional response. Figure 3(b) showed the projection view of OCT structural image, illustrating the well spatial registration [compare with Fig. 3(a)]. We observed that INS evoked a comparably localized, fOCT signal in rat cortex [images in Fig. 3(d) and quantified in Fig. 3(f)]. SOI 1 contained a maximal number of significant pixels (∼449  pixels) that spanned approximately the same lateral extent as the OISI activation, SOI 2 exhibited a reduced number of pixels (∼354  pixels), and SOI 3 exhibited no significant scattering response to INS. fOCT responses were consistent across trials (trial number n=15) and among different animals (animal number n=5). The fOCT signal mirrored the OISI signal with respect to spatial localization, averaged amplitude of all significant pixels, and the evoked time courses [compare Fig. 3(e) with Fig. 3(f)]. Furthermore, as shown in depth-resolved sequences [Fig. 3(g)], this temporal correspondence of fOCT signals was maintained up to a depth of 500  μm with no significant difference among cortical depths, consistent with the 300- to 600-μm penetration depth of INS at 1870-nm wavelength.^42^

### 3.2 Temporal Analysis

**Fig. 4:**
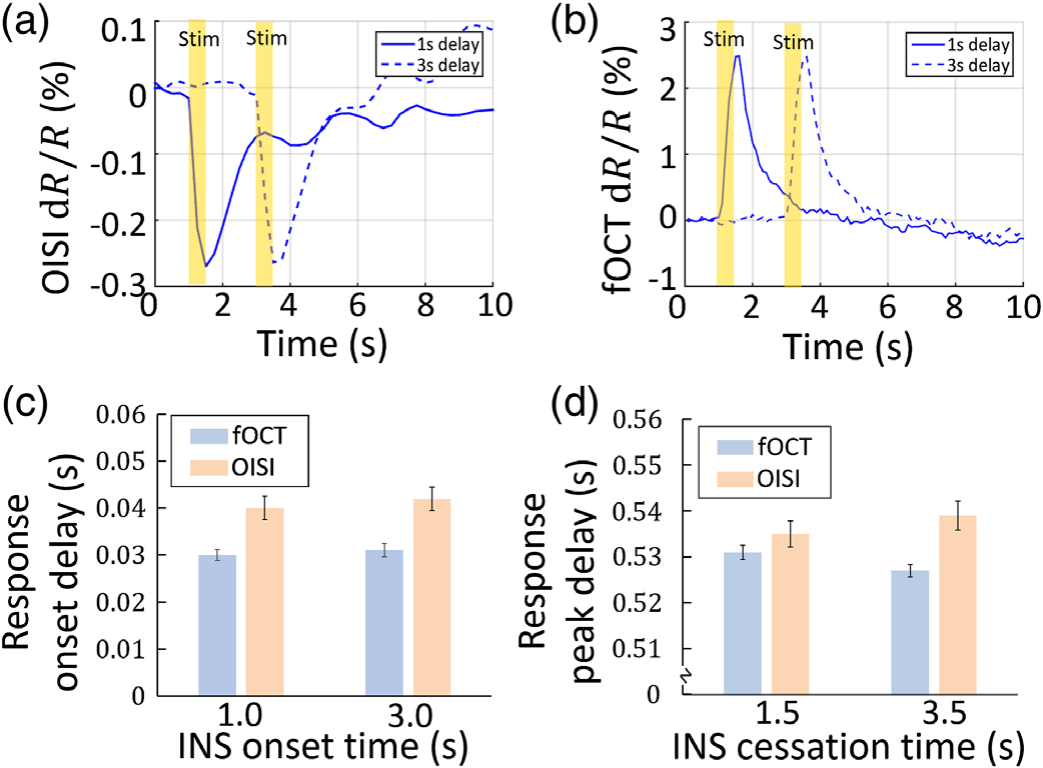
Temporal coincidence of INS-evoked fOCT signals. Time courses of (a) OISI and (b) fOCT of a representative rat, respectively. Temporal resolution is 17 and 4.2 ms for OISI and fOCT, respectively. Solid and dashed curves indicate a 1- and 3-s prestim period, respectively. Measurement of (c) the response onset delay and (d) the peak delay in fOCT and OISI, respectively. Averaged by 3×2.5  mm B-frame size in the x−z plane and 15 trials. Error bar: STD of five rats.

The fOCT signal was also temporally wedded to the INS stimulation. For Fig. 4, we averaged over all the pixels with significant changes in a representative rat. Two sets of trials with 1 and 3-s prestim period were performed. As shown in Fig. 4(a), the rise and the fall of the OISI signal are closely associated with the onset and cessation of INS, respectively. Similar temporal coincidence in the fOCT signals can be shown in Fig. 4(b). The onset delay of OISI, fOCT, and electrophysiological signal was defined as the time-point when the signal power increased larger than three times STD above the baseline after the start of INS. The peak delay was defined when the signal power reached the maximum after the start of INS. They are quantified in Figs. 4(c) and 4(d), respectively. The fOCT onset time delayed by ∼30  ms after INS, which was ahead of the OISI response for ∼10  ms. And the fOCT peak time delayed by ∼528  ms after INS, which was ahead of the OISI response for ∼5  ms.

### 3.3 Radiant Exposure Dependence

**Fig. 5:**
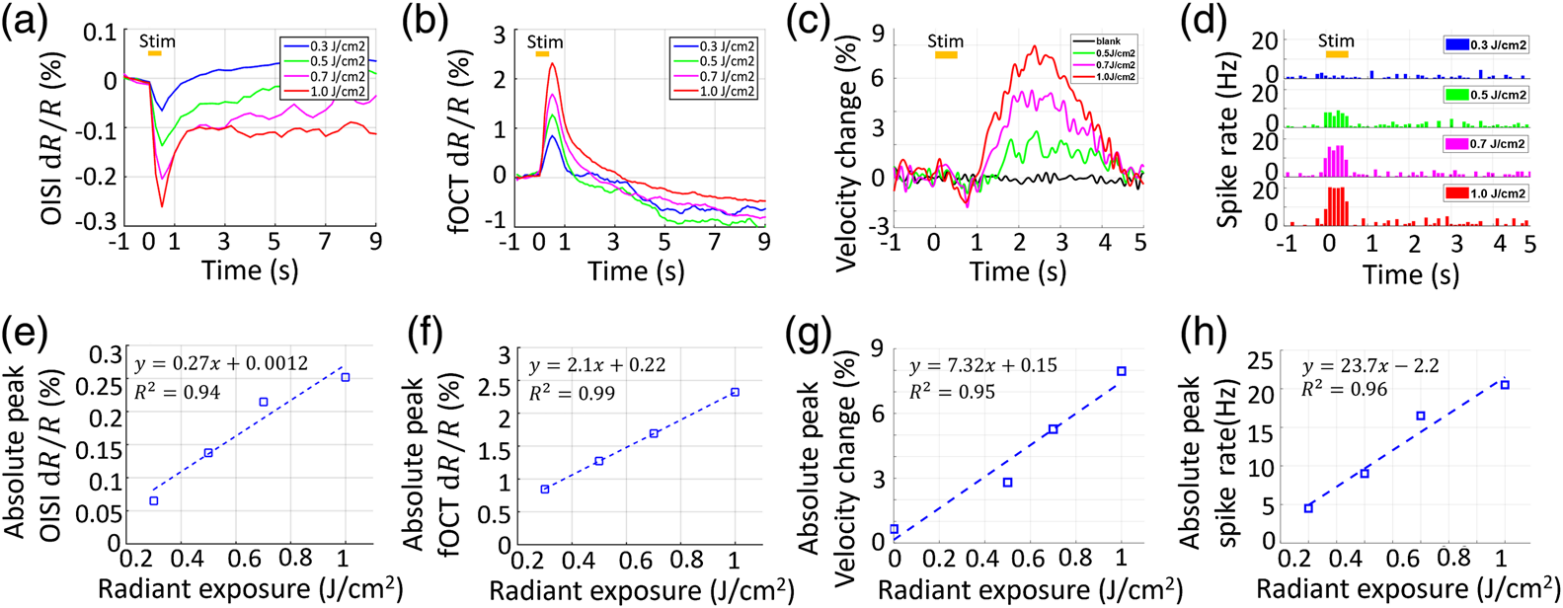
fOCT signal is positively correlated with INS radiant exposure. Time courses of (a) OISI and (b) fOCT signal for different radiant exposures. (c) The time course of flow velocity (derived from interframe decorrelation, 240 fps) in response to INS with radiant levels of 0 (blank), 0.5, 0.7, and $1.0\;J/{\mathrm{cm}}^{2}$. (d) Representative PSTH from a cortical layer with radiant levels of 0.5 and $1.0\;J/{\mathrm{cm}}^{2}$. Tungsten microelectrodes (∼1.3  MΩ) were inserted into the somatosensory cortex at depths of 50 to 750 and ∼500  μm away from the fiber tip. Threshold of spike was set to −41  mV. Radiant exposure versus peak amplitude of the (e) OISI fractional signal, (f) fOCT fractional signal, (g) velocity, and (h) peak spike rates.

Previous studies have shown that the fractional scattering signal would be linearly proportional to the membrane potential change.^15^ Figure 5 shows the response at different radiant exposures (0.3, 0.5, 0.7, $1.0\;J/{\mathrm{cm}}^{2}$ per pulse). As expected, increased INS radiant exposure led to an increase in the fOCT signal magnitude [see Fig. 5(b)], exhibiting a linear fit with radiant exposure [Fig. 5(f)]. OISI signal also presented a similar intensity dependence [see Figs. 5(a) and 5(e)], which is in agreement with previous optical imaging studies in rat cerebral cortex.^15^

Figure 5(c) shows the relative blood flow velocity changes in response to INS. The onset time of the velocity change was delayed by ∼1  s after INS, roughly consistent the measured ∼1  s in previous studies on cerebral RBC velocity response during rat forepaw electrical stimulation using functional ultrasound imaging,^52^ and those during hind paw electrical stimulation using the laser-Doppler flowmetry.^53^^,^^54^ The maximal velocity change was also correlated to radiant exposure as showed in Fig. 5(g).

To test the hypothesis that the fractional changes in OISI and fOCT were based on INS-evoked neuronal activities, electrophysiological recording was employed. As shown in Fig. 5(d), the increase of the spike rate during INS period (t=0 to 0.5 s) indicates excitatory activation, which was determined to be statistically significant in comparison to 500 ms pre-INS and 500 ms post-INS period ($p<6.1\times 10^{-6}$ and $p<3.7\times 10^{-4}$, paired two-tail t-test). The spike rate was correlated to the radiant exposure [see Fig. 5(d) and 5(h)]. The onset time of the spike rate was delayed by ∼4  ms when the bin range is set to be 1 ms.

## 4 Discussion and Conclusion

In this work, we proposed a label-free, all-optical INS-fOCT method for simultaneous neural manipulation and imaging. And we demonstrated that the fOCT signal is highly correlated to the INS-induced neural activations (confirmed by OISI and electrophysiology): the fOCT signal was reliable and regional and the area of fOCT signal corresponded with the INS-activated region (also the INS target area, see Fig. 3); the onset of fOCT signal was in synchrony with the INS onset (∼30  ms delay) and the peak was in synchrony with cessation times (∼528  ms delay, see Fig. 4); and the magnitude of fOCT signal exhibited a positive correlation with the INS radiant exposure (see Fig. 5). Because the light absorption of brain tissue and hemoglobin is negligible in comparison with the scattering at 1325-nm wavelength band,^55^ the INS-induced fOCT signal can be mainly attributed to the light scattering changes of brain tissue.

Both the neural and hemodynamic responses can contribute to the changes of light scattering. Although a vascular mask was used in this study to rule out the hemodynamic contribution to the derived fOCT scattering signals, the dilation of blood vessels may also lead to scattering changes in neighboring tissues and may have contributed to the final fOCT signals. However, there exists a time lag between the cellular activities and the neurovascular-coupled hemodynamic signals. The arteriole CBV-weighted signal has a lag of 1.8±0.2  s to the calcium signals, and the venule BOLD signal presents a lag of 2.3±0.2  s.^56^ In our study, the fOCT signal (peak time ∼528  ms delay) was earlier than the cellular calcium signal (peak time ∼716  ms delay) in Ref. 41, whereas the hemodynamic changes (onset time ∼1  s delay) was much latter than the fOCT signals (onset time ∼30  ms delay). Thus, although the origins of the slow temporal part (>∼1  s) is complex, the fast temporal part (<∼1  s) of fOCT signal most likely originates from the cellular activity directly. Additional investigations (e.g., using two-photon methods) are needed to further examine the relative contributions of cellular versus vascular components.

Furthermore, the fast temporal part of the fOCT signal might be induced by the changes in cell membrane ion channel orientations during action potentials, which lead to changes in local optical properties of neuronal membranes.^15^ According to this mechanism, the magnitude of the membrane potential change has a linear relationship with the induced light scattering changes, consistent with our observation that the fOCT signal is linearly dependent on the INS radiant exposure. The membrane voltage change has a small delay of ∼70  ms to the OCT scattering change in a single cultured Aplysia bag cell neuron,^23^ which is on the same order of magnitude as our measurement (fOCT onset time delayed ∼30  ms to INS, see Fig. 4). The differences in these two latencies may come from differences in stimulation methods or differences in signal propagation in the two cell types.^15^^,^^23^^,^^57^

The fast temporal part of the fOCT signal might also involve neuronal membrane displacements (or deformations) during stimulation, which has been detected with phase-sensitive interferometric or low-coherence imaging techniques.^24^^–^^26^^,^^58^^–^^61^ Typically, nanometer-scale displacements were detected on a millisecond time scale and were thought to be caused by the swelling and shrinkage of the nerve fiber^24^^,^^25^^,^^60^ and neural cell bodies.^58^^,^^61^ Akkin et al.^25^ reported that the nerve displacements accompanied fast light scattering changes. More work will be needed to understand the mechanism underlying the fOCT signals associated with the INS-evoked neuronal activities.

The proposed INS-fOCT has several advantages over the existing methods for simultaneously neural stimulation and recording. A combination of optogenetic actuator and optical calcium imaging enables selective stimulation and recording of genetically defined neurons in a spatiotemporal-specific manner.^3^^,^^7^^,^^62^ However, such a combined use is limited by several experimental challenges, including crosstalk between stimulation and imaging channels due to spectral overlap of the optogenetic probe and calcium indicator, and the limited stimulation and imaging depth due to strong visible light scattering within tissue. In addition, although optogenetics has been successfully applied in rodents, the requirement of viral transfection makes it less amenable to use in large animals such as nonhuman primates. Another approach for focal neural stimulation is optical activation of neurons bound with gold nanoparticles.^63^ Although the feasibility has been demonstrated in cultured neurons, biocompatibility of gold nanoparticles *in vivo* remains uncertain. In the proposed INS-fOCT, the stimulation and imaging channels work at distinct wavelengths (INS: 1870 nm, fOCT: 1325 nm), allowing simultaneous stimulation and imaging without risk of interchannel cross-talk. In addition, infrared working wavelengths enable a deeper penetration into brain tissue than with visible light.^55^ Furthermore, this method does not require contrast injection or viral transfection, making it compatible with non-human primate studies and potentially with human studies in clinical settings.

In conclusion, we demonstrate a label-free, all-optical INS-fOCT method for simultaneous neural manipulation and imaging. The fOCT signal is highly correlated to the INS-induced neural activations. Although further work is required to fully understand the origins of the fOCT signals, the fast component of the fOCT signal is most likely generated by the scattering property changes of cellular components in neural tissue. We believe the proposed INS-fOCT has numerous advantages for basic and clinical neuroscience research.
